# A Statistical Method for the Analysis of Speech Intelligibility Tests

**DOI:** 10.1371/journal.pone.0132409

**Published:** 2015-07-06

**Authors:** Wenli Hu, Brett A. Swanson, Gillian Z. Heller

**Affiliations:** 1 Department of Statistics, Macquarie University, Sydney, NSW, Australia; 2 Cochlear Ltd, Sydney, NSW, Australia; Beijing University of Posts and Telecommunications, CHINA

## Abstract

Speech intelligibility tests are conducted on hearing-impaired people for the purpose of evaluating the performance of a hearing device under varying listening conditions and device settings or algorithms. The speech reception threshold (SRT) is typically defined as the signal-to-noise ratio (SNR) at which a subject scores 50% correct on a speech intelligibility test. An SRT is conventionally measured with an adaptive procedure, in which the SNR of successive sentences is adjusted based on the subject's scores on previous sentences. The SRT can be estimated as the mean of a subset of the SNR levels, or by fitting a psychometric function. A set of SRT results is typically analyzed with a repeated measures analysis of variance. We propose an alternative approach for analysis, a zero-and-one inflated beta regression model, in which an observation is a single sentence score rather than an SRT. A parametrization of the model is defined that allows efficient maximum likelihood estimation of the parameters. Fitted values from this model, when plotted against SNR, are analogous to a mean psychometric function in the traditional approach. Confidence intervals for the fitted value curves are obtained by parametric bootstrap. The proposed approach was applied retrospectively to data from two studies that assessed the speech perception of cochlear implant recipients using different sound processing algorithms under different listening conditions. The proposed approach yielded mean SRTs for each condition that were consistent with the traditional approach, but were more informative. It provided the mean psychometric curve of each condition, revealing differences in slope, i.e. differential performance at different parts of the SNR spectrum. Another advantage of the new method of analysis is that results are stated in terms of differences in percent correct scores, which is more interpretable than results from the traditional analysis.

## Introduction

Measuring speech intelligibility in noise is an important endeavor in the clinical management of hearing loss. It can be used to assess the benefit a person receives from a hearing aid or cochlear implant, and to track their performance over time. It is also used in the research and development of hearing devices, to compare the effectiveness of alternative sound processing algorithms.

A common approach is to play a pre-recorded sentence, mixed with noise, to the subject, who attempts to verbally repeat it. The clinician then records a score for the sentence based on the number of words that the subject repeated correctly. Alternatively, scores can be based on only the key words in the sentence, or based on morphemes, a linguistic unit (for example, in the sentence “He hits the ball”, the word “hits” contains two morphemes, “hit” and “s”). A subject is typically tested with a list of 10 to 32 sentences taken from a corpus of sentences compiled for this purpose [1] [2] [3] [4].

**Fig 1:**
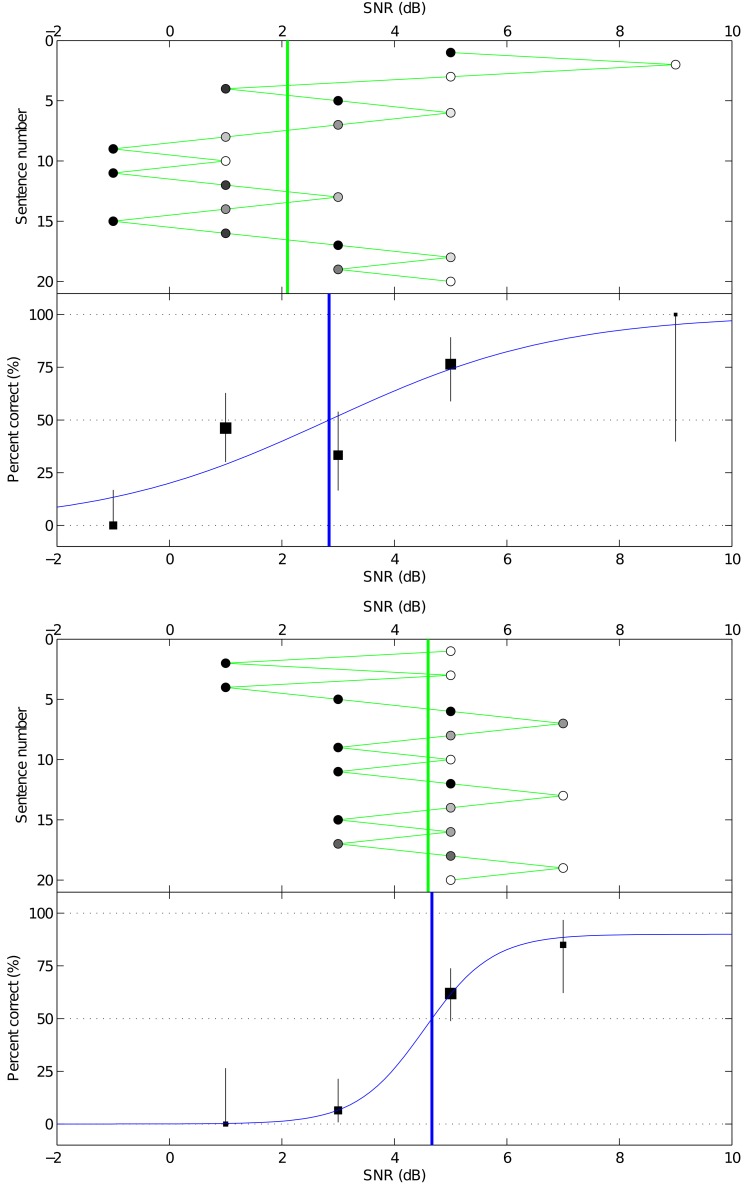
Two example adaptive tracks and psychometric curves from study one. Each part of the figure shows a track of 20 sentences for subject B1, corresponding to Table 1. The top panel of each part shows the adaptive track, with sentence number running down the page; each sentence is represented by a circle, with its horizontal location indicating the SNR, and its gray-scale fill indicating the score, with 100% correct as white, and 0% as black. The green vertical line shows the SRT estimate obtained by averaging the SNRs of the final 16 sentences. The bottom panel of each part shows the mean percent correct score at each SNR, with the size of each square proportional to the number of sentences that were presented at that SNR, and a confidence interval calculated according to the binomial distribution. It also shows the fitted psychometric curve, and the blue vertical line indicates the corresponding SRT estimate [4].

Speech in noise tests are sometimes performed at a fixed signal-to-noise ratio (SNR), to give a percent-correct measure of intelligibility. If the test is repeated at different SNRs, and the scores are plotted as a function of SNR, the resulting curve is known as a psychometric function that is typically S-shaped, for example the logistic function [5]. When designing a study into the effects of a sound processing algorithm, the differences in performance between subjects can be so large that testing all subjects at the same SNR would be prone to floor or ceiling effects. An alternative is to measure the speech reception threshold (SRT) of each subject, which is typically defined as the SNR at which the subject scores 50% correct. The SRT is conventionally estimated by an adaptive procedure, in which the SNR of each sentence is adjusted based on the subject’s previous responses. Adaptive threshold estimation methods were initially developed for experiments in which the subject provides a binomial response on each trial [6]; for example, identifying the correct interval in an *N*-alternative forced-choice test. These methods can readily be applied to sentence tests; if the subject correctly identifies more than half of the words in the sentence, then the SNR is reduced (making the next sentence more difficult), and conversely if the subject correctly identifies less than half of the words, then the SNR is increased (making the next sentence easier). With an appropriate adaptive rule, the SNR should converge to the SRT [3]. Two example adaptive tracks are shown in Fig 1.

The SRT can be estimated as the mean of the SNR levels, excluding some initial sentences [1] [3]; or by the mean of the SNR levels at the turns, where a turn (or reversal) is defined as a trial in which the adaptive rule changed direction [6]; or by fitting a psychometric function [7] [4]. A set of SRT estimates is usually analyzed using a simple statistical method such as a t-test or repeated measures analysis of variance (ANOVA).

**Fig 2:**
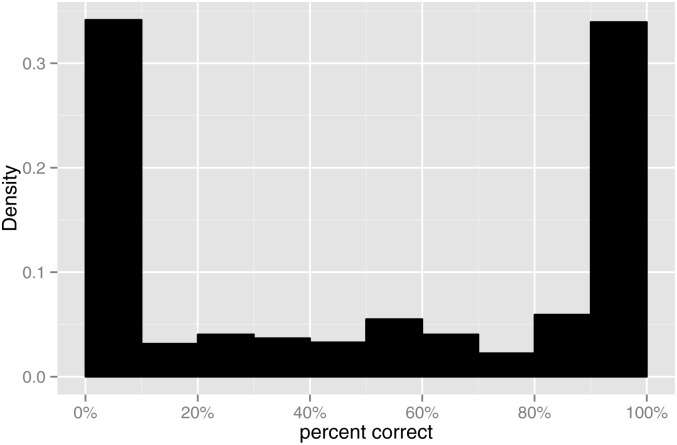
Study One: Histogram of percent correct.

Although Dawson et al. [4] found that fitting a psychometric function provided the best SRT test-retest reliability, there are some limitations in this approach. Occasionally, a subject’s average scores are not a monotonically increasing function of SNR; an example is shown in Fig 1. This could be due to random fluctuation, or a lapse in the subject’s concentration, or a run of more difficult sentences (despite efforts to equalise sentence difficulty [4]). Such cases can produce a poor fit. Furthermore, the fitting method assumed a binomial distribution [5], but the assumption that a sentence containing *K* words consists of *K* independent Bernoulli trials is violated, because recognition of one word is not independent of the other words. Sentences representative of everyday conversation have contextual cues, meaning that if the subject recognises the first few words, then they are more likely to recognise the remaining words. At the SRT, although the average word score is 50%, it is relatively uncommon to score near 50% for any particular sentence; instead some sentences receive scores near 100%, and a roughly equal number of sentences receive scores near 0%. A histogram of the sentence scores for Study One (described in the next section), with large spikes at values 0% and 100%, is shown in Fig 2.

Regardless of the method used to calculate the SRT, summarizing an entire adaptive track by a single number suffers from a loss of information. Applying repeated measures ANOVA to a set of SRT estimates implicitly assumes that the psychometric functions for the different conditions are of similar shapes, differing only in the SRT values, and differences in the slope of the psychometric function or its asymptotic value are ignored. We propose an alternative approach, a zero-and-one inflated beta regression model, in which an observation is a single sentence score rather than an SRT. This model makes fewer assumptions about the data and provides more valuable information.

## Materials and Methods

### The speech perception data sets

The two studies described below were approved by the Human Research Ethics Committee of the Royal Victorian Eye & Ear Hospital, Melbourne, and each subject provided written informed consent.

The new statistical method was applied to data from two studies involving Nucleus cochlear implant recipients. The two studies shared a number of characteristics. Both studies used a repeated-measures design, in which each subject served as their own control, and the aim was to compare performance with different sound processing algorithms under one or more listening conditions. The two studies administered an adaptive SRT test, using the Australian Sentence Test in Noise (AuSTIN) [4]. The target speech was presented at 65 dB SPL, and the level of the interfering noise was adjusted based on the subject’s responses. Morpheme scoring was used. Each adaptive track used a list of 20 sentences, and the SRT was calculated as the mean of the SNRs of the final 16 sentences.

#### Study One

The first study compared the speech recognition of seven bilateral cochlear implant recipients in the presence of an interfering talker. The first factor of interest was the sound processing algorithm (“algorithm”). The details of the sound processing algorithms are not relevant to the statistical analysis of the results, so the three algorithms are simply labelled “A”, “B” and “C”, and the question was whether the three algorithms yielded differences in the subjects’ performance. The second factor was the direction of the interfering talker (“noise direction”), which was either from the front (“F”) or from both sides (“S”). As the target speech was presented from the front, it was hypothesized that performance would be better for side interferers, as subjects could potentially use the difference in spatial location to segregate the two voices. The third factor was the gender of the interfering talker (“noise gender”). As the target voice was female, it was hypothesized that performance would be better for a male interferer, as subjects could potentially use the difference in voice pitch to segregate the two voices. For the noise direction and noise gender factors, interaction with the algorithm factor would indicate that the sound processing algorithms differed in their effectiveness in conveying spatial or pitch cues. The SRT for each subject was measured four times (i.e. four adaptive tracks, totalling 80 sentences), for each of the 12 conditions (3 algorithms × 2 noise directions × 2 noise genders). The adaptive rule used a 4 dB step size for the initial four sentences and a 2 dB step size for the remaining sentences.

#### Study Two

The second study [8] compared speech intelligibility as a function of a single factor, the sound processing algorithm (“algorithm”), consisting of a standard algorithm (“Beam”) and five variants of a spatial noise reduction algorithm, labeled “SpS0”, “SpZ-3”, “SpZ0”, “SpZ+3”, “SpZ+6” (again, the details of the sound processing algorithms are not relevant here). Twelve subjects participated. The target speech was presented from the front, while the noise consisted of four interfering talkers, each presented from a separate loudspeaker in the rear half-circle, with locations that changed from sentence to sentence. The SRT for each subject was measured twice (i.e. two adaptive tracks, totalling 40 sentences), for each of the six algorithms. The adaptive rule was the same as in the first study, with the exception that the SNR for the fifth sentence was equal to the average of the SNRs of the initial four sentences and the SNR at which the fifth sentence would have been presented in response to the score of the fourth sentence [3]. The primary hypothesis was that the spatial noise reduction algorithms would give better performance than Beam, with a secondary goal to determine which variant of spatial noise reduction gave the best performance.

### Traditional Approach

The traditional approach used SRT as the response variable in ANOVA models. A single observation was therefore the SRT calculated over a track of 20 sentences. In both studies, every subject was evaluated across all factors, and repeated measures ANOVA was applied. The underlying assumption of sphericity was assessed by Mauchly’s test of sphericity. The Greenhouse-Geisser adjustment was applied to adjust the degrees of freedom in case of violation of the sphericity assumption. The significance level was set as 0.05. If the main effect was found to be significant, multiple comparisons were performed subsequently to generate inferences.

### The Statistical Model of The New Approach

In this approach, a single observation is a sentence. The response variable *y* in the statistical model is the proportion of morphemes correctly identified, i.e. *y* = *r*/*N*, where *N* is the number of morphemes in a sentence and *r* is the number of morphemes correctly identified. A binomial model may seem an obvious choice for *y*, but clearly the independence assumption of the binomial model is not met, due to context effects. As *y* is a proportion, a promising probability model is the beta distribution:

$$f_{1}\left(y;\alpha ,\beta \right)=\frac{1}{B(\alpha ,\beta )}y^{\alpha -1}{\left(1-y\right)}^{\beta -1}\;\;y\in (0,1)$$ (1)

which has mean *E*(*y*) = *α*/(*α*+*β*). It is advantageous, in regression modeling, for the response distribution to be expressed in a parametrization in which the mean is a parameter. We therefore base our modeling on the following alternative parametrization of the beta distribution [9]:

$$\begin{array}{r} f_{2}\left(y;\mu ,\sigma \right)=\frac{1}{B\left(\frac{\mu \left(1-\sigma^{2}\right)}{\sigma^{2}},\frac{\left(1-\mu \right)\left(1-\sigma^{2}\right)}{\sigma^{2}}\right)}y^{\frac{\mu \left(1-\sigma^{2}\right)}{\sigma^{2}}-1}{\left(1-y\right)}^{\frac{\left(1-\mu \right)\left(1-\sigma^{2}\right)}{\sigma^{2}}-1}\;y\in \left(0,1\right)\\ 0<\mu <1;\;0<\sigma <1 \end{array}$$ (2)

which has the advantage that *E*(*y*) = *μ*. We have *Var* (*y*) = *σ* ^2^ *μ*(1−*μ*), and the parameters *μ* and *σ* are connected with the original parameters *α* and *β* in Eq (1) with relations *α* = *μ*(1−*σ* ^2^)/*σ* ^2^ and *β* = (1−*μ*)(1−*σ* ^2^)/*σ* ^2^. A feature of the beta distribution is that the endpoints *y* = 0 and *y* = 1 are inadmissible. If the data had small frequencies at either endpoint, with most observations lying in the interior of (0, 1), this could be accommodated by scaling *y* to lie in the interior of (0, 1). However in our data we observe high frequencies at zero (no morphemes recognized) and one (all morphemes correctly identified). This feature is accommodated by the zero-and-one-inflated beta distribution [10], which has parameters *p*_0_ and *p*_1_ for probability spikes at zero and one, respectively. We write this as a mixed discrete-continuous probability function:

$$f_{3}\left(y;\mu ,\sigma ,p_{0},p_{1}\right)=\{\begin{array}{cc} p_{0} & y=0\\ \left(1-p_{0}-p_{1}\right)\cdot f_{2}(y;\mu ,\sigma ) & y\in (0,1)\\ p_{1} & y=1 \end{array}$$ (3)

which has overall mean *E*(*y*) = (1−*p*_0_−*p*_1_)*μ*+*p*_1_. However, we need to reparametrize once more, because in estimating parameters of the probability Eq (3), we have to respect the constraint 0 < *p*_0_ + *p*_1_ < 1, which is awkward to achieve numerically. In addition, parameter estimates ${\hat{p}}_{0}$ and ${\hat{p}}_{1}$ are negatively correlated, which is not a good property. We use instead

$$f_{4}(y;\mu ,\sigma ,\nu ,\tau )=\{\begin{array}{cc} \frac{\nu }{1+\nu +\tau } & y=0\\ \frac{1}{1+\nu +\tau }\cdot f_{2}(y;\mu ,\sigma ) & y\in (0,1)\\ \frac{\tau }{1+\nu +\tau } & y=1 \end{array}$$

where *ν* > 0 and *τ* > 0. The probability masses of measures 0 and 1 are associated with the two shape parameters *ν* and *τ* through the relations *ν* = *p*_0_/(1−*p*_0_−*p*_1_) and *τ* = *p*_1_/(1−*p*_0_−*p*_1_).

The four parameters *μ*, *σ*, *ν* and *τ* are modeled with covariates, as well as random effects to account for within-subject correlation:

$$\begin{array}{c} \text{log}\left(\frac{\mu }{1-\mu }\right)=x^{⊤}\beta +u_{1}\\ \text{log}\left(\frac{\sigma }{1-\sigma }\right)=z^{⊤}\gamma +u_{2}\\ \text{log}(\nu )=h^{⊤}\lambda +u_{3}\\ \text{log}(\tau )=k^{⊤}\rho +u_{4} \end{array}$$ (4)

where *x*, *z*, *h* and *k* are vectors of known covariates, which may be overlapping or distinct; *β*, *γ*, *λ* and *ρ* are corresponding coefficient vectors; and $u_{j}∼𝓝(0,\delta_{j}^{2}),j=1,\ldots ,4$ is a random effect for subject. Logit links are used for the parameters constrained to (0, 1), i.e. *μ* and *σ*, and log links for those constrained to ℝ^+^ (*ν* and *τ*), as is common practice in generalized linear modeling. Parameter estimation is achieved in the R package gamlss, in which up to four distribution parameters may be modeled simultaneously [11, 12], using maximum (penalized) likelihood estimation. Model selection was based on the Generalized Akaike Information Criterion (GAIC).

Although parameters *ν* and *τ* determine the probability masses for proportion correct at zero and one, the probabilities *p*_0_ and *p*_1_ are not modeled with regression structures directly, and the effect of the covariates on these probabilities is difficult to interpret. For given covariate values *h* and *k*, fitted values for proportion correct equal to zero (${\hat{p}}_{0}$) and one (${\hat{p}}_{1}$) are derived algebraically, with random effects ${\hat{u}}_{j}$ assumed to be zero:

$$\begin{array}{l} {\hat{p}}_{0}=\frac{\hat{\nu }}{1+\hat{\nu }+\hat{\tau }}=\frac{\text{exp}(h^{⊤}\hat{\lambda })}{1+\text{exp}(h^{⊤}\hat{\lambda })+\text{exp}\left(k^{⊤}\hat{\rho }\right)}\\ {\hat{p}}_{1}=\frac{\hat{\tau }}{1+\hat{\nu }+\hat{\tau }}=\frac{\text{exp}(k^{⊤}\hat{\rho })}{1+\text{exp}(h^{⊤}\hat{\lambda })+\text{exp}\left(k^{⊤}\hat{\rho }\right)}\;. \end{array}$$

To facilitate interpretation, fitted probabilities enhanced with confidence intervals are plotted against the covariates. The confidence intervals are based on the parametric bootstrap [13]. Specifically, given estimates $\hat{\beta }$, $\hat{\gamma }$, $\hat{\lambda }$ and $\hat{\rho }$, and for each combination of covariate values *x*, *z*, *h*, *k*, the parameter estimates $\hat{\mu }$, $\hat{\sigma }$, $\hat{\nu }$ and $\hat{\tau }$ are computed, using Eq (4) and assuming ${\hat{u}}_{1}=\cdots ={\hat{u}}_{4}=0$:

$$\hat{\mu }=\frac{\text{exp}(x^{⊤}\hat{\beta })}{1+\text{exp}(x^{⊤}\hat{\beta })}\;;\;\hat{\sigma }=\frac{\text{exp}(z^{⊤}\hat{\gamma })}{1+\text{exp}(z^{⊤}\hat{\gamma })}\;;\;\hat{\nu }=\text{exp}\left(h^{⊤}\hat{\lambda }\right)\;;\;\hat{\tau }=\text{exp}\left(k^{⊤}\hat{\rho }\right)\;.$$

These estimates are then used to generate pseudo observations (bootstrap samples) from model *f*_4_. Five hundred such bootstrap samples are generated, each of which contains the same number of observations under every combination of covariate values, as in the original data. Each sample is fitted with zero-and-one inflated beta regression, and the fitted overall means of proportion correct are obtained. Endpoints of the 95% confidence intervals are computed as the lower and upper 2.5% percentiles of the fitted proportions correct, at each covariate combination.

## Results

### Study One

An excerpt of the data from the first study is shown in Table 1, and the full dataset is given in S1 Data. It shows the scores for two sentence lists of 20 sentences each, for the same subject and listening condition. Corresponding plots are shown in Fig 1. For the purpose of the traditional analysis, the 40 sentence scores in Table 1 are aggregated to two SRT estimates, given in Table 2.

**Table 1:** An excerpt of the sentence scores from study one. The table shows two adaptive tracks (tracks 2 and 7), each of 20 sentences, for subject B1. Each row of the table contains data for one sentence. Each sentence score is specified by the number of morphemes repeated correctly (*num*_*correct*), and the number of morphemes in the sentence (*num*_*items*).

subject	algorithm	track	noise_dir	noise_gender	speech_level	snr	num_correct	num_items
B1	C	2	S	F	65	5	0	6
B1	C	2	S	F	65	9	4	4
B1	C	2	S	F	65	5	7	7
B1	C	2	S	F	65	1	1	7
B1	C	2	S	F	65	3	0	5
B1	C	2	S	F	65	5	7	8
B1	C	2	S	F	65	3	3	6
B1	C	2	S	F	65	1	5	7
B1	C	2	S	F	65	-1	0	7
B1	C	2	S	F	65	1	7	7
B1	C	2	S	F	65	-1	0	6
B1	C	2	S	F	65	1	1	6
B1	C	2	S	F	65	3	4	6
B1	C	2	S	F	65	1	3	6
B1	C	2	S	F	65	-1	0	7
B1	C	2	S	F	65	1	1	6
B1	C	2	S	F	65	3	0	5
B1	C	2	S	F	65	5	5	6
B1	C	2	S	F	65	3	2	5
B1	C	2	S	F	65	5	7	7
B1	C	7	S	F	65	5	7	7
B1	C	7	S	F	65	1	0	6
B1	C	7	S	F	65	5	6	6
B1	C	7	S	F	65	1	0	6
B1	C	7	S	F	65	3	0	6
B1	C	7	S	F	65	5	0	6
B1	C	7	S	F	65	7	3	6
B1	C	7	S	F	65	5	4	7
B1	C	7	S	F	65	3	0	6
B1	C	7	S	F	65	5	6	6
B1	C	7	S	F	65	3	0	5
B1	C	7	S	F	65	5	0	6
B1	C	7	S	F	65	7	7	7
B1	C	7	S	F	65	5	4	6
B1	C	7	S	F	65	3	0	7
B1	C	7	S	F	65	5	4	7
B1	C	7	S	F	65	3	2	7
B1	C	7	S	F	65	5	2	6
B1	C	7	S	F	65	7	7	7
B1	C	7	S	F	65	5	6	6

**Table 2:** An excerpt of the SRT data from study one. In the traditional approach, the 40 sentence scores in Table 1 are reduced to the two SRT estimates shown here.

subject	algorithm	track	noise_dir	noise_gender	speech_level	SRT
B1	C	2	S	F	65	2.1
B1	C	7	S	F	65	4.6

#### Traditional Approach

A three-way repeated measures ANOVA was used and no violation of the sphericity assumption was found. With respect to main effects, algorithm and noise direction were not significant. The noise gender factor, however, was significant, with estimated marginal group means of SRT of 2.59 dB for male interferers, and 3.99 dB for female interferers. The interaction term of noise gender and algorithm was also significant, and the summary is given in Table 3.

**Table 3:** Tests of within-subjects effects in study one.

Effect	Type III SS	F value	P value
algorithm	4.096	0.543	0.584
noise_gender	165.060	50.490	0.000
noise_dir	0.771	0.179	0.676
algorithm×noise_gender	34.576	7.131	0.002
algorithm×noise_dir	11.520	2.059	0.137
noise_gender×noise_dir	2.555	1.633	0.212
algorithm×noise_dir×noise_gender	1.001	0.380	0.686

Effects of algorithm were investigated by dividing the data into two subsets, with male and female interferers separately. With female interferers, the effect of algorithm was significant (*F*_2,54_ = 4.676, *p* = 0.013), and pairwise comparisons with Bonferroni adjustments suggested that algorithm A had significantly better (lower) SRT than algorithm B (*p* = 0.021), with estimated marginal mean difference of -0.918 dB. No other comparisons were significant. With male interferers, the effect of algorithm, however, was not significant (*F*_2,54_ = 1.444, *p* = 0.245).

Effects of noise gender were also investigated by splitting the data by algorithm. The noise gender factor was significant for each algorithm (A: *F*_1,27_ = 5.019, *p* = 0.033; B: *F*_1,27_ = 29.096, *p* < 0.001; C: *F*_1,27_ = 28.331, *p* < 0.001), suggesting that SRT estimates were significantly better (lower) for male interferers than for female. No other terms were significant.

#### Proposed Approach

Parameter estimates for the modeling of *μ*, *σ*, *ν* and *τ* are given in Table 4. No covariates were significant for *σ*. For *μ*, *ν* and *τ*, SNR, noise gender and algorithm were all significant. In addition, noise gender-algorithm interaction was significant for *μ* and *τ*; SNR-algorithm interaction was significant for *ν*; and SNR-noise gender interaction was significant for *τ*.

**Table 4:** Modeling of *μ*, *σ*, *ν* and *τ* in Study One.

Coefficients		Estimate	Std. Error	t-value	P-value
parameter	*μ*				
link	logit				
Intercept		-0.184	0.054	-3.406	<0.0001
snr		0.068	0.007	9.580	<0.0001
noise_gender					
F		—	—	—	—
M		-0.020	0.066	-0.305	0.760
algorithm					
A		—	—	—	—
B		-0.036	0.066	-0.542	0.588
C		-0.168	0.063	-2.687	0.007
noise_gender×algorithm					
M:B		0.184	0.092	1.995	0.046
M:C		0.213	0.089	2.406	0.016
parameter	*σ*				
link	logit				
Intercept		-0.302	0.020	-15.280	<0.0001
parameter	*ν*				
link	log				
Intercept		1.129	0.083	13.556	<0.0001
snr		-0.290	0.022	-13.198	<0.0001
noise_gender					
F		—	—	—	—
M		-0.315	0.058	-5.414	<0.0001
algorithm					
A		—	—	—	—
B		-0.335	0.102	-3.283	0.001
C		-0.366	0.105	-3.489	<0.001
snr×algorithm					
snr:B		0.119	0.028	4.312	<0.0001
snr:C		0.025	0.029	0.871	0.384
parameter	*τ*				
link	log				
Intercept		-0.866	0.098	-8.819	<0.0001
snr		0.241	0.016	14.790	<0.0001
noise_gender					
F		—	—	—	—
M		0.262	0.132	1.984	0.047
algorithm					
A		—	—	—	—
B		-0.179	0.098	-1.815	0.070
C		-0.579	0.010	-5.808	<0.0001
noise_gender×algorithm					
M:B		0.132	0.138	0.961	0.336
M:C		0.389	0.138	2.810	0.005
snr×noise_gender					
snr:M		-0.029	0.022	-1.298	0.194

**Fig 3:**
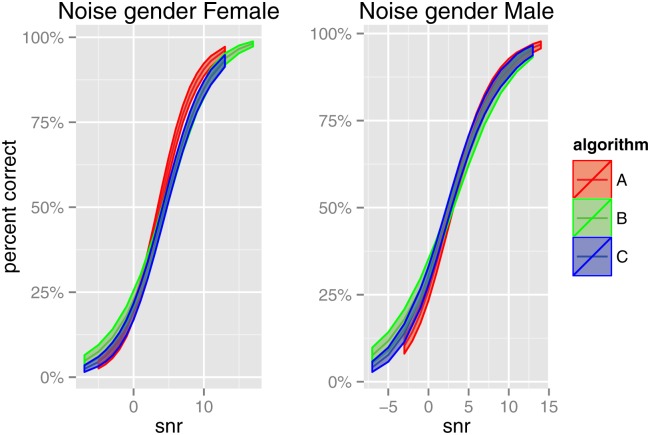
Study One: Fitted overall means of percent correct, showing effect of algorithm. 95% confidence intervals were calculated using 500 parametric bootstrap samples.

**Fig 4:**
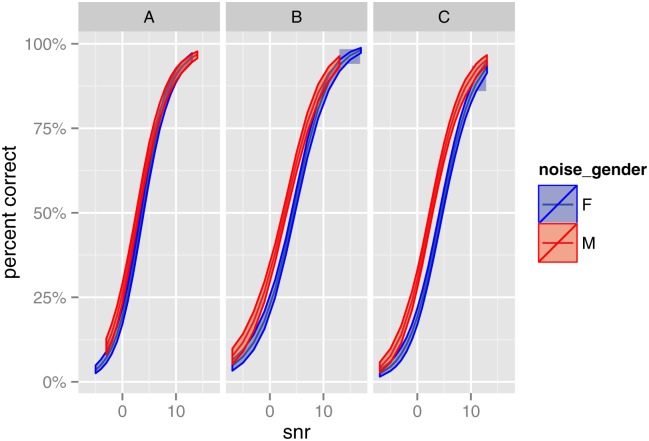
Study One: Fitted overall means of percent correct, showing effect of noise gender. 95% confidence intervals were calculated using 500 parametric bootstrap samples.

The fitted overall means of percent correct (i.e. $(1-{\hat{p}}_{0}-{\hat{p}}_{1})\hat{\mu }+{\hat{p}}_{1}$) for each algorithm are shown in Fig 3, separately for both noise genders, together with confidence intervals constructed by parametric bootstrap. The curves for algorithms B and C largely overlap at all SNRs, indicating little difference between those algorithms. However, the curve for algorithm A has a steeper slope than those for algorithms B and C; for female interferers, the three curves overlap at low SNRs, but start to separate at higher SNRs. This difference in slope between algorithms was not detected in the traditional approach. Similarly, Fig 4 presents the fitted value curves and confidence intervals, separately for each algorithm, showing the effect of noise gender. For all three algorithms, the male interferer provided significantly better speech intelligibility than the female, with the difference being larger for algorithms B and C.

**Fig 5:**
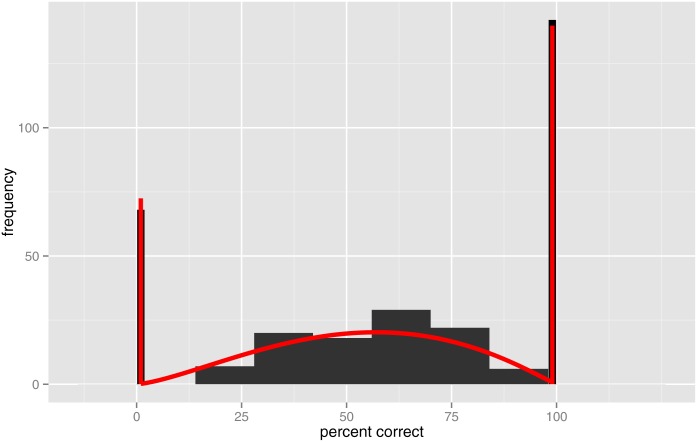
Study One: Observed frequencies, and fitted zero-and-one inflated beta distribution. Algorithm = A, gender = Female, SNR = 5 (312 subjects).

The fitted zero-and-one inflated beta distribution is shown in Fig 5 for the subset of subjects having algorithm = A, gender = Female, and SNR = 5 (*n* = 312).

### Study Two

#### Traditional Approach

The dataset is given in S2 Data. Following Hersbach et al. [8], a one-way repeated measures ANOVA was used, in which marginal violation of the sphericity assumption was found (*p* = 0.048) and the Greenhouse-Geisser adjustment was applied to the degrees of freedom of F-statistics involved. The algorithm factor was significant (*F*_3.457,79.506_ = 47.401, *p* < 0.001). More specifically, the estimated means of SRT were 0.171 dB, -2.583 dB, -3.279 dB, -3.938 dB, -4.446 dB and -3.987 dB for Beam, SpS0, SpZ-3, SpZ0, SpZ+3 and SpZ+6 respectively. Pairwise comparisons with Bonferroni adjustments showed that Beam had significantly worse SRTs than all five variants of the spatial noise reduction algorithm (*p* < 0.001). In addition, SpS0 had significantly worse SRTs than SpZ0, SpZ+3 and SpZ+6 (*p* = 0.008, *p* < 0.001, *p* = 0.005 respectively). Pairwise comparisons among the four variants of SpZ-3, SpZ0, SpZ+3 and SpZ+6 suggested no significant differences.

#### Proposed Approach

**Fig 6:**
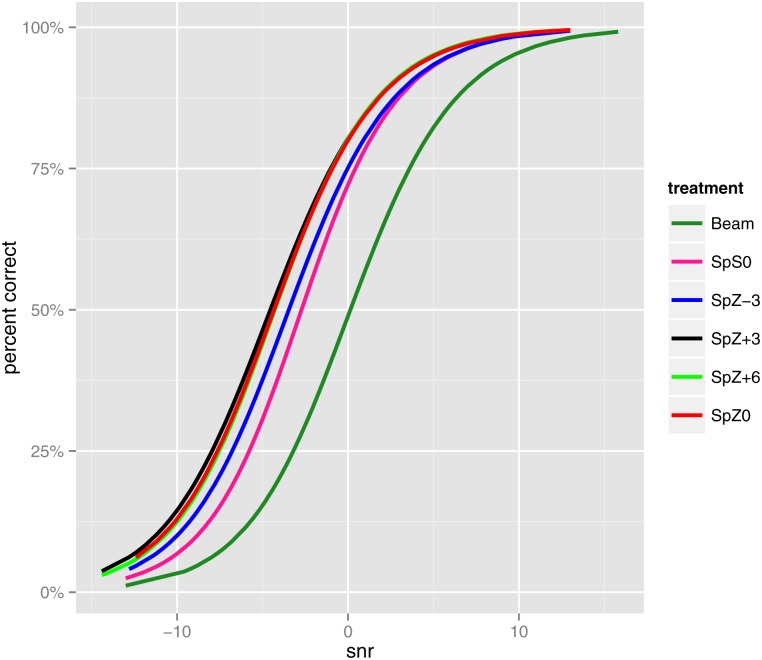
Study Two: Fitted overall means of percent correct.

Parameter estimates for the modeling of *μ*, *σ*, *ν* and *τ* are given in Table 5. No covariates were significant for *σ*. Fig 6 shows the fitted overall means of percent correct, i.e. the mean psychometric functions for the six algorithms. All curves appear to have the same slope. A horizontal line at 50% correct intercepts each psychometric function at an SNR equal to its SRT, illustrating the 4.6 dB SRT improvement of SpZ+3 over Beam, as found in the traditional approach.

**Table 5:** Modeling of *μ*, *σ*, *ν* and *τ* in Study Two.

Coefficients		Estimate	Std. Error	t-value	P-value
parameter	*μ*				
link	logit				
Intercept		0.071	0.052	1.369	0.171
snr		0.071	0.005	14.634	<0.0001
algorithm					<0.0001
Beam		—	—	—	—
SpS0		0.200	0.078	2.566	0.010
SpZ-3		0.236	0.075	3.125	0.002
SpZ+3		0.391	0.076	5.110	<0.0001
SpZ+6		0.365	0.078	4.705	<0.0001
SpZ0		0.336	0.076	4.396	<0.0001
parameter	*σ*				
link	logit				
Intercept		-0.333	0.024	-14.040	<0.0001
parameter	*ν*				
link	log				
Intercept		-0.479	0.097	-4.950	<0.0001
snr		-0.289	0.009	-30.838	<0.0001
algorithm					<0.0001
Beam		—	—	—	—
SpS0		-0.560	0.139	-4.042	<0.0001
SpZ-3		-1.028	0.142	-7.260	<0.0001
SpZ+3		-1.388	0.144	-9.623	<0.0001
SpZ+6		-1.170	0.144	-8.148	<0.0001
SpZ0		-1.251	0.143	-8.739	<0.0001
parameter	*τ*				
link	log				
Intercept		-0.624	0.096	-6.490	<0.0001
snr		0.254	0.009	29.143	<0.0001
algorithm					<0.0001
Beam		—	—	—	—
SpS0		1.009	0.136	7.443	<0.0001
SpZ-3		0.943	0.137	6.876	<0.0001
SpZ+3		1.092	0.141	7.752	<0.0001
SpZ+6		1.193	0.139	8.569	<0.0001
SpZ0		1.160	0.138	8.384	<0.0001

**Fig 7:**
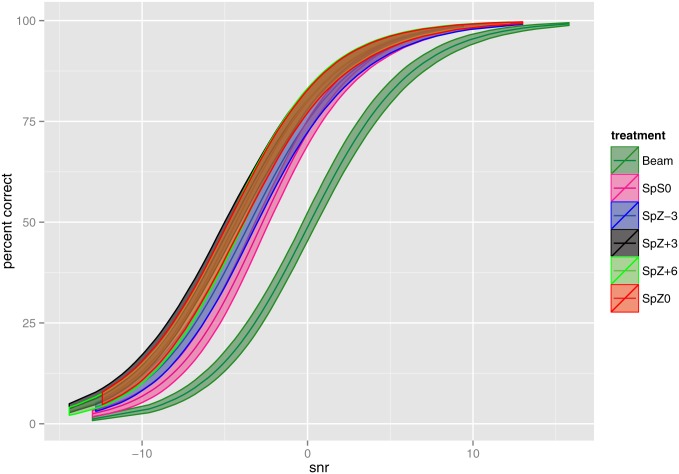
Study Two: Fitted overall means of percent correct, with confidence intervals. 95% confidence intervals were calculated using 500 parametric bootstrap samples.

Confidence intervals for the mean psychometric functions, obtained by parametric bootstrap, are presented in Fig 7, demonstrating that Beam had significantly lower speech intelligibility scores than all five spatial noise reduction algorithm variants. The curves for SpZ0, SpZ+3, and SpZ+6 overlap, suggesting that their performances can be viewed as indistinguishable. The curve for SpS0 is separated from the curves of SpZ0, SpZ+3 and SpZ+6, indicating a significant difference.

## Discussion and Conclusions

The traditional approach has two stages: firstly, an SRT estimate is computed for each adaptive track; and secondly, a linear model is applied to the set of SRT estimates. The first stage, which distils a set of sentence SNR levels and scores into a single number, the SRT estimate, discards much of the available information. For example, the within-subject, within-condition variability is measured by the spread of four SRT values in study one, and two SRT values in study two. The corresponding set of sentence scores is a potential source of information regarding this variability, but is ignored. A psychometric fit can provide an estimate of the slope of the psychometric function, with shallower slopes implying more variability in SRT estimates, but there is no obvious means of incorporating slopes into the traditional approach.

In contrast, the proposed approach applies a generalized linear model to the entire set of sentence scores, utilizing all available information. It can provide the mean SRTs of each condition, as in the traditional approach, but is more informative as it also provides estimates of the entire mean psychometric function for each condition.

One limitation of a retrospective analysis of the data is that the adaptive rule used in these studies started with a high SNR, then adjusted the SNR towards the 50% correct point. This concentrates the observations near the 50% correct point, which is the most efficient placement for estimating the SRT [6] but yields relatively few observations at lower SNRs. This makes differences at the extremes of the SNR spectrum difficult to detect. If the goal of a study is to estimate the entire mean psychometric function, then a different adaptive rule should be used. One solution is to randomly interleave multiple adaptive tracks, each targeting different percent correct scores, e.g. 30% and 70% correct [6] [7]. This is readily handled by the proposed approach.

The most practical benefit of the proposed approach is that it allows the difference between two conditions to be expressed in terms of percent correct scores. For example, in study two, the traditional approach states that the best spatial noise reduction algorithm gave a 4.6 dB SRT benefit over Beam. However, terms such as decibels and SRTs are unfamiliar to most cochlear implant recipients. Instead, the proposed approach allows the result to be better understood: in a noisy situation, average scores improved from 25% correct with Beam to 62% with the best spatial noise reduction algorithm.

## Supporting Information

S1 Data Study One data. StudyOne.xlsx is the full data set (as in Table 1), StudyOne_srt.xlsx is the data set summarized as SRT (as in Table 2).
(ZIP)

S2 Data Study Two data. StudyTwo.xlsx is the full data set, StudyTwo_srt.xlsx is the data set summarized as SRT.
(ZIP)
